# Study on the efficacy and safety of the combination of Shi’s manual therapy and percutaneous endoscopic lumbar diskectomy for lumbar disc herniation with radiculopathy: study protocol for a multicenter randomized controlled trial

**DOI:** 10.1186/s13063-022-06195-y

**Published:** 2022-04-23

**Authors:** Huihao Wang, Weian Yuan, Zhongxiang Yu, Xiang Wang, Xinxin Zhao, Zhen Deng, Guangyue Yang, Weinan Chen, Zhibi Shen, Hongsheng Zhan

**Affiliations:** 1 Shi’s Center of Orthopedics and Traumatology (Institute of Traumatology, Shuguang Hospital), Shuguang Hospital Affiliated to Shanghai University of Traditional Chinese Medicine, Shanghai, 201203 China; 2 Tongji University School of Medicine, Shanghai, 200065 China; 3 Shanghai Baoshan Hospital of Integrated Traditional Chinese and Western Medicine, Shanghai, 201999 China

**Keywords:** Lumbar disc herniation with radiculopathy, Shi’s manual therapy (SMT), Percutaneous endoscopic lumbar discectomy (PELD), Multicenter randomized controlled trial, Protocol

## Abstract

**Background:**

Lumbar disc herniation (LDH) is a common chronic musculoskeletal disorder that seriously affects quality of life. The percutaneous endoscopic lumbar diskectomy (PELD) technique was developed to address spinal nerve root compression through direct visualization of pathological findings while minimizing tissue destruction upon exposure. It is an effective and safe treatment for LDH. However, recurrent LDH is a major concern after lumbar discectomy for primary LDH. A considerable number of clinical studies have reported that patients with LDH with radiculopathy could benefit from manual therapy. Shi’s manual therapy (SMT) was established based on traditional Chinese medicine (TCM) theory and has been shown to have a superior effect in alleviating muscle tension and loosening joints to improve lumbar and leg pain, radiculopathy, stiffness, activity discomfort, and related disorders. However, there is a lack of high-quality clinical evidence to support this conclusion. The purpose of this study is to evaluate the efficacy and safety of the combination of Shi’s manual therapy (SMT) and PELD for LDH with radiculopathy.

**Methods/design:**

A multicenter randomized controlled trial (RCT) with a 1-year follow-up period will be performed. A total of 510 participants with LDH with radiculopathy will be recruited from four clinical centers. The sample size was estimated, and statistical analysis will be performed and supervised by biostatisticians from an independent third-party research institution. Two hundred fifty-five subjects will be randomly allocated to each group. The subjects in the control group will undergo PELD. Participants in the intervention group will be treated with a combination of SMT and PELD. Recurrence rate is the primary endpoint and the survival analysis of recurrence rate is the secondary endpoint, and the primary analysis of recurrence rate is the chi-square test and the secondary analysis of recurrence rate is survival analysis. The primary outcome measure is the recurrence rate of LDH with radiculopathy at the 1-year follow-up after treatment. The secondary outcome measures will be the ODI score, the VAS score for pain for the lumbar spine and lower limbs, the straight leg raise angle, the stability of the operated lumbar segment, and the SF-36 scores. Assessments will occur at baseline, postoperation, and 1 week, 4 weeks, 13 weeks, 26 weeks, and 1 year postoperation. In addition, adverse events related to clinical symptoms and signs and the results of laboratory tests will be documented during the clinical trials.

**Discussion:**

This study will provide reliable evidence of the effectiveness and safety of the combination of SMT and PELD for LDH with radiculopathy. If the results are favorable, it is expected that patients with LDH with radiculopathy will benefit from this study, and many patients could gain a good alternative treatment for LDH with radiculopathy.

**Trial registration:**

China Registered Clinical Trial Registration Center ChiCTR2000036515. Registered on 13 November 2020.

## Background

Herniated lumbar discs involve the displacement of disc material (nucleus pulposus or annulus fibrosis) beyond the intervertebral disc space [1], and the overall prevalence rate of this condition is 70% in developed countries [2]. The diagnosis can be confirmed by radiological examination. However, MRI findings of herniated discs are not always accompanied by clinical symptoms [3]. Conservative treatments are first-line treatments for lumbar disc herniation (LDH), including medication, bed rest, physical therapy, massage, kinesitherapy, manual therapy, and traction therapy. Manual therapy related to clinical massage, spinal manipulation (SM), or mobilization is applied to release the joint fixation of the involved segment and to restore the flexibility of the lumbar spine to improve the biomechanical environment of the affected nerve roots in LDH patients. A review reported that SM is considered an alternative or complementary medical method, although with limited curative effect, and only better than placebo if there is no severe adverse effect in its performance [4]. In the North American Spine Society’s (NASS) Evidence-Based Clinical Guideline for the Diagnosis and Treatment of LDH with radiculopathy, spinal manipulation is the grade of recommendation: C. There is an insufficient evidence to make a recommendation for or against the use of spinal manipulation compared with chemonucleolysis in patients with lumbar disc herniation with radiculopathy, and the grade of recommendation is I (insufficient evidence) [5].

Despite a lack of clear evidence-based medicine of its safety and efficacy, spinal manipulation is still popular in clinical treatment. A considerable number of clinical studies have reported that patients will benefit from SM practice for LDH with radiculopathy patients. Previous reports proved that effective SM will load appropriate stress on the involved joints and produce nonnoxious stimuli [6, 7]. The benefits are accompanied by potential yet rare risks in terms of serious adverse events. Therefore, it is reasonable to use an “appropriate mechanical intervention” to achieve a balance between releasing the tension of the joint and reducing the risk of injury during SM performance.

Surgical intervention may become necessary if conservative therapeutic measures fail or paralysis is present. Minimally invasive spine surgery has become more common. The percutaneous endoscopic lumbar diskectomy (PELD) technique was developed to address spinal nerve root compression by direct visualization of pathological findings while minimizing tissue destruction upon exposure. It is an effective and safe treatment for LDH, lumbar spinal stenosis, recurrent LDH, and other lumbar diseases. Operative times, blood loss, lengths of hospitalization, and need for postoperative pain medications have all generally been reduced with microendoscopic discectomy [8]. However, complications related to PELD include dural tears (1.1%), intervertebral infections (0.47%), incomplete removal of the disc (2.77%), nerve root injury, recurrence, and soon [9]. Recurrent LDH is a major concern after lumbar discectomy for primary LDH and occurs in approximately 10.3–17.6% of patients [10, 11]. The clinical characteristics and risk factors for recurrent LDH, such as sex, obesity (body mass index ≥25), older age (≥50 years old), and smoking history, remain controversial [12, 13].

Shi’s manual therapy (SMT) was established based on the TCM theory that lumbar and leg complaints are induced by the tendon off-position and joint subluxation (International Classification of Diseases 11th Revision, ICD-11). It is one of the characteristic techniques of Shi’s traumatology TCM academic school with years of clinical experience summary and accumulation, and the details will be described below. Techniques involving rapid loading of stress are also called “manipulation” or “thrusting adjustment” and are characterized by high velocity and low amplitude (HVLA). According to advanced research progress and our clinical observation, there is no obvious discomfort effect in the short term, and there has been no report of a severe adverse event in the short and middle term for SMT [14]. The main purpose is to alleviate muscle tension and loosen joints to improve uncomfortable symptoms, such as lumbar and leg pain, radiculopathy, stiffness, activity discomfort, and related disorders.

In clinical practice, we found that the organic combination of SMT and PELD can significantly reduce the recurrence rate of LDH with radiculopathy; however, there is no high-quality evidence thus far. Therefore, the authors designed an integrated cooperative treatment protocol. If our hypothesis is correct, it will provide a choice for patients with LDH with radiculopathy who prefer no surgery or an alternative and complementary option before receiving PELD. The participants will fully understand the benefits and risks before being recruited and signing the informed consent form.

To the authors’ knowledge, a detailed description of a multicenter RCT of integrated cooperative management with SMT and PELD for LDH with radiculopathy is not available in the related literature.

### Study aims

This study is a multicenter RCT study aimed at investigating the efficacy and safety of the combination of SMT and PELD for LDH with radiculopathy. In terms of effectiveness, we mainly focus on the advantages of SMT combined with PELD, compared with PELD alone, in reducing the recurrence rate.

## Methods/design

### Study setting

A multicenter prospective RCT with unblinded treatment and blinded outcome assessment will be conducted in four clinical centers in Shanghai, China, including Shuguang Hospital affiliated with Shanghai University of Traditional Chinese Medicine (TCM), Shanghai Municipal TCM Hospital affiliated with Shanghai University of TCM, Tenth People’s Hospital affiliated with Tongji University and Shanghai Baoshan Hospital of Integrated TCM.

#### Sample size calculation

Sample size estimation was performed, and the statistical analysis will be performed and supervised by biostatisticians from the Clinical Epidemiology and Medical Statistics Center, Tongji University School of Medicine, as an independent institution.

The recurrence rate of LDH with radiculopathy at 1 year postoperation will be the primary outcome measure for sample size calculations, as reported by a previously published trial at 17.6% [11]. Pearson’s chi-square test was used to compare the rates of the two groups after treatment. Sample size calculation formula:$n=N=\frac{{\left({\mathrm{U}}_{\upalpha}+{\mathrm{U}}_{2\upbeta}\right)}^2\ast 2P\left(1-P\right)}{{\left({P}_1-{P}_0\right)}^2}$ The positive rate *P1* of the test group is (1–8.6%), the positive rate *P0* of the control group is (1–17.6%), and *P* is the average positive rate =0.025, 1 − *β* = 0.80.

The recurrence rate in the intervention group (effect size =8.6; standard deviation = 2.5) will be reduced by 9% compared with that in the control group (effect size = 17.6; standard deviation = 2.5). In addition, a dropout rate of 16% will also be taken into account. The estimations indicated that 255 individuals per group were required. Hence, a minimum of 510 patients will be necessary for the sample size.

### Randomization

This trial is designed as a multicenter, randomized, and controlled study. This clinical trial is a multicenter trial that is conducted simultaneously in four hospitals. The central randomization system uses dynamic block randomization and competitive enrollment methods. The trial design has two points. Level factors were divided into two levels according to the severity of the disease: (a) ODI index ≤ 30 points and (b) ODI index > 30 points. Two levels according to age: (a) < 60 years old and (b) ≥ 60 years old. All subjects were randomly enrolled in the dynamic zone group.

The central stochastic system weight setting is as follows (random order): (1) Stratification factors; the first layer: the degree of illness: (a) ODI index ≤ 30 points; (b) ODI index> 30 points; the second layer: age: (a) < 60 years old; (b) ≥60 years old; (2) center weight (research center); (3) grouping factors (groups A and B).

Thus, the number of project blocks is *x*, and each block contains n subjects. Randomized allocation is carried out by competition entry. Therefore, each center first allocates a number of blocks, that is, the number of allocated cases for each center is determined by the sponsor and the research institution. The investigator’s institution negotiates whether to continue to allocate a number of blocks to the center, and the process will continue until all block allocation is completed; if a center cannot complete all enrollment due to insufficient recruitment of suitable subjects, etc. The statistician and the statistic party negotiate whether to allocate cases between centers.

An independent statistician will perform the subject enrollment and intervention assignment. After suitable participants are filtered by the acceptance criteria, their basic information will be transmitted to the statistician. He will produce a computer-generated randomization sequence that will be placed in sequentially numbered opaque sealed envelopes. The randomization sequence will contain equal numbers of participants in each group. Due to the nature of the interventions, the participants were not blinded to the treatment group. To minimize bias randomization, concealed allocation, specific inclusion and exclusion criteria, blinded outcome assessment, blind data analysis, and intention-to-treat analysis have been used.

**Fig. 1:**
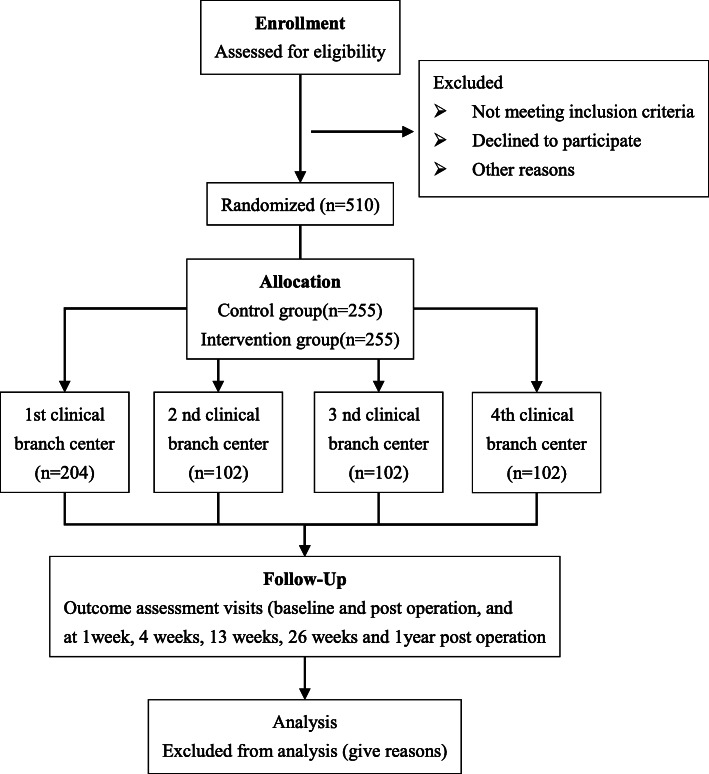
Study flow diagram of trial participation

Two hundred fifty-five participants with LDH with radiculopathy will be randomly allocated to each group. The number of cases allocated to each center is provided in Fig. 1.

### Ethics approval

The study design and procedures were approved by the IRB of Shuguang Hospital Affiliated to Shanghai University of TCM (Approval Number: 2020-850-57-01), and protocol version 2.1/20210120 is currently active. Protocol version 1.0/20200821 was revised to include urine HCG testing of women of childbearing age during subject screening. The procedures followed the Declaration of Helsinki from 1975, as revised in 2000.

### Patient and public involvement

Five hundred ten participants with LDH with radiculopathy will be recruited in the four research centers through official hospital websites, offline advertising, and project participants’ self-media recruitment advertisements from October 2020 to September 2022 and by referral from an orthopedic surgeon and a physiotherapist in every center. The number of subjects was sufficient to support the successful completion of the project according to the number of related patients in recent 5 years in the four research centers. A flow chart of trial participation is provided in Fig. 1. The flowchart for participant identification, inclusion, study design, interventions, assessments, and follow-up are shown in Table 1. Personal information about potential and enrolled participants will be collected by outpatient doctors. The outcome data will be retained in the custody of the independent statistician, and all data will be aggregated and maintained by the supervising doctor to protect confidentiality after the trial.

**Table 1:** Flowchart of enrollment, intervention, data collection, and follow-up

Timepoint	Screening/Visit 0 (0~3 days before surgery)	Visit 1 (The 2nd day after surgery)	Visit 2 (1 week ± 2 days after surgery)	Visit 3 (4 weeks ± 5 days after surgery)	Visit 4 (13 weeks ± 1 week after surgery)	Visit 5 (48 weeks ± 2 weeks after surgery)	Visit 6 (1 year ± 2 months after surgery)
**Enrollment**							
*[Eligibility screen]*	*×*						
*[Informed consent]*	**×**						
*[Random allocation]*	**×**						
**Assessments**							
*[VAS scores]*	**×**	**×**	**×**	**×**	**×**	**×**	**×**
*[ODI]*	**×**	**×**	**×**	**×**	**×**	**×**	**×**
*[Straight leg raising angle]*	**×**	**×**	**×**	**×**	**×**	**×**	**×**
*[SF-36v2]*	**×**					**×**	**×**
*[Positive and lateral X ray]*	**×**	**×**		**×**	**×**	**×**	**×**
*[Dynamic X ray]*	**×**				**×**	**×**	**×**
*[CT and MRI]*	**×**	**×**				**×**	**×**
**Adverse events**		**×**	**×**	**×**	**×**	**×**	**×**

The supervising doctor will have access to the final trial dataset and disclosure of contractual agreements that limit such access for investigators.

#### Inclusion criteria

(1) 20 to 70 years old. (2) Met the diagnostic criteria of lumbar disc herniation with radiculopathy by Tian Wei et al. [15]. (3) ODI ≥ 10. (4) The course of the condition is more than 3 months and has not improved with conservative therapy for more than 6 weeks. (5) Voluntarily participated in this trial and signed the informed consent form.

#### Exclusion criteria

(1) Tuberculosis, cancer, severe osteoporosis, acute infectious diseases, acute suppurative inflammation, severe cardiovascular, cerebrovascular, liver, kidney, hematopoietic, digestive system diseases, or mental disorders. (2) Definite lumbar segmental instability or developmental lumbar spinal stenosis. (3) Current pregnancy or is prepared to get pregnant. (4) Received other treatment at the same time. (5) Those who participated in other clinical trials within 3 months.

#### Withdrawal criteria

(1) The researcher considered unsuitable for participating in the trial, (for example, unable to correctly understand the questionnaires, inconvenient to follow-up for living in other provinces). (2) The subjects disobeyed the trial plan and combined with other drugs. 3) Serious adverse reactions. (4) The patients’ complaints continued to increase, which proved that trial participation was not suitable.

Those who meet 1 of the above conditions should be rejected.

### Intervention measures

#### Control group

Participants in the control group will undergo PELD in 0~3 days.

#### Intervention group

Participants in the intervention group will be treated with SMT in 0~3 days, and then the PELD procedure will proceed.

Emergency medication: If the subject's pain is intolerable, he can take emergency painkillers (diclofenac sodium sustained release tablets 75 mg qd), provided uniformly when necessary, and record the dose of the drugs.

#### Percutaneous endoscopic lumbar diskectomy (PELD)

A senior physiotherapist with medical doctorate qualifications and a minimum of 10 years of experience will operate. PELD procedures will be performed as followed [16]:

1. Anesthesia and positioning: PELD by the transforaminal approach is performed under local anesthesia (20 ml 1% lidocaine and 20 ml normal saline) with the patient in the lateral position or prone position on a radiolucent operating table
2. Surgical technique: Axial MRI or CT is used to obtain an approximate idea of the distance of the skin entry point from the midline. The needle trajectory is planned to target the ruptured fragment while avoiding the contents of the peritoneal sac. After satisfactory anesthesia with the patient, the surgeon routinely performs routine disc puncture and staining and then expands the working channel step by step according to Young's technique (YESS) and the Maxmore technique. C-arm X-ray machine fluoroscopy confirms that the pipeline is placed in a good position. Then, the pipeline is connected to the irrigation device and the light source, the surgeon sequentially performs ventral, dorsal, cranial, and caudal bony and soft decompression with laser and forceps on the patient's target intervertebral disc segment, and the contralateral and lateral recesses are gradually decompressed. After that, the surgeon observes the pulsation state of the nerve root of the dural sac, cough test, and straight leg raising test to evaluate the degree of spinal canal decompression until satisfaction. Finally, the surgeon draws out the irrigation fluid in the patient's intervertebral disc space, injects steroid drugs (1 ml of Depot), sutures the wound, and completes the operation
3. Postoperative management of the patients will include (a) a simple adhesive bandage was on the stitch, and the suture will be removed after 10~14 days; (b) wearing of a lumbar brace will be required for 6 weeks, and avoidance heavy physical work and sitting for long periods will be required for 3 months; and (c) oral Celebrex capsule qd will be used as needed for 0~3 days

#### Shi’s manual therapy (SMT)

A senior physiotherapist with medical doctorate qualifications and a minimum of 25 years of experience in SMT will perform the interventions on the participants. Participants in the intervention group will be treated with SMT for 0~3 days, and the straight leg raising angle will be evaluated. If the patient’s straight leg raise angle on the affected side is close to that of the healthy side, the treatment is finished; if not, the PELD procedure will proceed.

The key procedures of SMT include the following steps:

1. Anesthesia and positioning: SMT is performed under local anesthesia (20 ml 1% lidocaine and 20 ml normal saline) with the patient at the target disc segment nerve root outlet in the supine position on a radiolucent operating table
2. Bending hips and knees, pressing and stretching techniques: the patient lies in the supine position. The therapist presses both lower limbs with hips and knees, pulls and stretches the affected leg in various directions several times, and then rotates left and right to pull and stretch the affected leg several times
3. Straight leg raises and compression techniques: the assistant presses the patient’s normal hip joint, the therapist presses the knee with one hand and the ankle with the other, lifts the patient’s lower limbs, gradually raising it to 80°~ 90°, repeating 10~15 times. Then, the therapist stands on the normal side of the patient and raises the patient’s legs in a back-holding style. The therapist holds the patient’s feet with his hands over his head and puts the therapist’s back against the patient’s knee joint to prevent flexion. After the therapist raises the patient’s affected limb to the limit range, foot extension exercises are performed passively 3~5 times
4. Lumbar spine oblique pulling technique in lateral position: the patient lies on the affected side, and the therapist straightens the patient’s lower limbs and flexes the hips and knees of the upper limbs. Then, the therapist presses the patient’s shoulder joint outward and pushes down, while the sacroiliac joint is fixed and presses inwardly. It is advisable to hear a “click” sound from the waist. It is the same procedure that will be used on the other side. The treatment will be stopped if the patients feel uncomfortable or for any other reasons, but the recorded data will be analyzed statistically

### Assessment and follow-up

Follow-up assessments with questionnaires will be conducted and will include the ODI, the VAS score for pain of the lumbar spine and lower limbs, the straight leg elevation angle of the affected side, and the SF-36 health survey. Pre- and postoperative magnetic resonance imaging (MRI), computed tomography (CT), and X-ray films will be documented. The assessments will be performed by 2 physicians who are not involved in the treatments.

Participants will receive a serial preoperative radiographic evaluation, with routine anteroposterior (AP), lateral and dynamic radiographic views, MRI, and CT scans to determine the target level in conjunction with a thorough clinical history and physical examination. Then, diagnostic selective nerve root blocks or electromyography (EMG) and nerve conduction studies will be assessed to determine the individual’s radiculopathy origins [9].

The first examination will be conducted before the operation for baseline assessment (visit 0). Then, assessments will be conducted on the second day postoperation (visit 1) and at 1 week (visit 2), 4 weeks (visit 3), 13 weeks (visit 4), 26 weeks (visit 5), and 1 year (visit 6) postoperation. The primary and secondary outcomes and possible complications will be recorded. All participants will complete the ODI questionnaire and VAS for pain for the lumbar spine and lower limbs. In addition, CT, MRI, and X-ray imaging, including AP, lateral, and dynamic (hyperextension and hyperflexion) radiographic view assessments, will be conducted. The radiation exposure dose is safe for all participants and will be assessed by two radiologists. Note that dynamic X-ray imaging will be carried out at visit 0, visit 4, visit 5 and visit 6, while MRI and CT scans will be carried out at visit 0, visit 1, visit 5, and visit 6.

The project team will conduct regular follow-up calls and text messages and set up a health club to prevent subjects from being lost to follow-up. The participants will be followed up when the treatment ceases.

### Outcome measures

#### Primary outcome

1. Recurrence rate

The outcome index is the recurrence rate of LDH with radiculopathy at 1-year postoperation in the two groups. Clinically speaking, herniation at the same level and on the same side would be more appropriate for recurrence. If the symptom returns after a pain-free period, it will be considered a recurrence [17].

#### Secondary outcomes

##### Recurrence rate and Oswestry Disability Index (ODI)

The Simplified Chinese-Mandarin Chinese version of the ODI will be applied to evaluate the degree of low back pain and disability [18], which was the primary endpoint.

##### The severity of lumbar and lower limb pain (VAS)

The VAS has also been used in many studies to measure pain as the main complaint [19] and has shown high reliability and validity. Low back pain and lower limb pain will be evaluated with a 10-point scale: 0 as painless and 10 as extremely painful. The patients will be asked to provide an average pain level over the previous 24 h.

##### Straight leg raising angle of the affected side

A protractor will be used to measure the straight leg angle of the healthy side and the affected side to assess the efficacy of the treatment and to calculate the difference on both sides. The straight leg raise test, also called the Lasegue test, is a fundamental neurological maneuver performed during physical examinations of patients with lower back pain and is aimed at assessing sciatic compromise due to lumbosacral nerve root irritation [20].

4) Stability of the lumbar spine

Radiological parameters will be measured, including lumbar curvature (L1-S1, tangential method) in the neutral position with the Harrison posterior tangent method and the segmental Cobb angle of hyperextension and hyperflexion, on X-ray films at the operative level (SA) [21]. Radiographic instability is defined as evidence of translational motion at one spinal motion segment more than 3 mm in the lumbar spine or 5 mm at L5-S1 or as angulation of one motion segment more than 10° on lateral flexion-extension radiographs [22].

5) Short Form 36 version 2 (SF-36v2) Health Survey scores

We used the Singapore English and Chinese language version of the SF-36v2 questionnaire to document health-related quality of life. The version includes 36 self-administered questions that examine 8 dimensions of the participant’s general health, including physical functioning, physical roles, bodily pain, general health, vitality, social functioning, emotional roles, and mental health, with higher scores (range, 0–100) reflecting better-perceived health [23].

### Adverse events (AE)

AEs related to clinical symptoms and signs and the results of laboratory tests will be documented during the clinical trials and reported to the supervising doctors. Expected AEs included the subject’s worsening symptoms, recurrence, nerve damage, infection, abnormalities in liver and kidney function, abnormal electrocardiogram, etc. The doctor will use a mixed approach to collect harms and reports in publications. The harms will be reported to other relevant parties (e.g., IRB, data monitoring committee).

After the expert committee has determined that the subject has indeed suffered damage related to this research, the sponsor will bear corresponding responsibilities in accordance with national laws and regulations and provide corresponding compensation or compensation for damage related to the test.

If the subject combines the treatment and examination required for other diseases at the same time, it will not be included in the free scope.

### Quality assurance

To ensure that interventions are of a high standard and delivered following the trial protocol, surgeons and physiotherapists responsible for performing SMT and PELD will attend a 2-day training workshop on the delivery of the intervention programs. They will also be provided with a written protocol and standardized recording documents. In addition, all interventions provided to patients will be carefully recorded.

A monitor will be set up throughout the study to monitor the quality of the study. Each research center will set up a clinical research coordinator (CRC) to assist researchers in conducting research.

### Data analysis

#### Statistical methods

The primary and secondary outcome measures are either continuous or ordinal and will be analyzed using generalized linear mixed models. Recurrence rate is the outcome index, and survival analysis is the secondary index. Subgroup analysis was performed according to ODI index (secondary outcome index) and age. The predictors will be time, treatment group, and an interaction term for time by treatment group. The primary analysis of recurrence rate is the chi-square test and the secondary analysis of recurrence rate is survival analysis. The effects of these protocols will be tested through both intention-to-treat and per-protocol analyses. For the intention-to-treat analysis, missing posttreatment or follow-up outcome data will be replicated from previous measures available (by assuming no change for noncompleters). For the per-protocol analysis, data from excluded subjects will be disregarded for the analysis. The groups will be compared at baseline by the chi-square test for qualitative data and by the *t* test for quantitative data. To analyze changes in outcomes at baseline, posttreatment, and follow-up between and within groups, analysis of variance with repeated measures will be applied. A normality test will be applied to the outcome measures; when data are not normally distributed, equivalent nonparametric tests will be used. The primary and secondary outcome measures will also be compared between treatment groups at each time point using independent *t* tests. The results will be presented as percentages for categorical variables and as the means, medians, standard deviations, and 95% confidence intervals for continuous variables. Data analyses will be performed using SPSS 22.0 for Windows version 18.0 (SPSS Inc., Chicago, IL, USA), and the significance value for all tests will be set at *P* < 0.05.

There will be no interim analyses and the principal investigator will receive the data from CRC every month and have the right to make the final decision to terminate the trial.

#### Controlling bias

It should be noted that the number of subjects assigned to Shuguang Hospital affiliated with Shanghai University of TCM is double that of the other centers. Shuguang Hospital is divided into two independent branches, with different doctors, inpatient departments, and operating rooms, so it can be regarded as two subcenters. Therefore, the number of subjects in all subcenters is balanced.

It was not possible to blind the surgeons and therapists to the interventions.

The independent statistician will audit the trial conduct every 12 weeks and report it directly to the superior doctor.

## Discussion

This protocol outlines the rationale and design for a multicenter RCT to investigate the safety of the combination of SMT and PELD compared with PELD only for LDH with radiculopathy and the superiority in reducing the recurrence rate in 6 months.

The key factor affecting the quality of this trial is the quality of the SMT and PELD given by the therapists and surgeons from the 4 clinical centers. Even though doctors may perform the same SMT and PELD techniques, it is understandable that the operational details and personal habits might be different. The potential differences will be minimized through the following:

1. Before officially starting, every research participant in this project needed to obtain good clinical practice (GCP) certificates authorized by the China SFDA. It is to ensure that all aspects of research are standardized and to reduce bias during the implementation process.
2. To make the operation process as standardized and consistent as possible, videos of the procedures were recorded by a professional videographer for SMT performed by a senior therapist and for PELD performed by an experienced surgeon in Shuguang Hospital. All SMT therapists and PELD surgeons in the 4 clinical centers have to be trained and pass the assessment.
3. Only one senior SMT therapist and one PELD surgeon will be appointed to perform the interventions in one clinical center. None of them will be allowed to perform the intervention until they have been trained and meet the requirements of this project.

It should be noted that Shuguang Hospital affiliated with Shanghai University of TCM is divided into two independent branches that include different doctors, inpatient departments, and operating rooms, enabling it to be regarded as two subcenters. Therefore, the number of subjects in all subcenters is balanced.

Shi's orthopedics and traumatology academic school with manual therapy is well known throughout China, with a history of 150 years, and it is officially recognized by China’s health care administration, which is extremely helpful for improving patient compliance in the study. However, the risk associated with spinal manipulation still needs to be noted. The subjects will be eligible for inclusion and exclusion criteria to minimize the occurrence of complications. Otherwise, the surgeon doctor will immediately perform PELD or routine open surgery once the patient has adverse reactions after SMT, such as nerve root damage or paralysis.

Statistical analysis is another challenging problem that is difficult to resolve. A multicenter prospective RCT will be conducted in four clinical centers, and 255 participants will be randomly allocated to each group. Participants in the intervention group will be treated with SMT first, and the straight leg raising angle will be evaluated. However, we assume that if the patient’s straight leg raise angle on the affected side is close to that of the healthy side, then the treatment is complete; if not, the PELD procedure will be performed. The data will be calculated and analyzed through hierarchical statistics by an independent biostatistician.

This design of the protocol has an advantage in the expertise of the physiotherapist. However, this may affect the generalizability of the conclusion. The results will contribute to evidence-based manual therapy, leading to improved clinical decision-making in this field of clinical practice.

The original participant-level data or any other documents will be uploaded to and monitored by the clinical research integration platform (CRIP)-Electronic Data Capture (EDC) System of Shanghai Shenkang Hospital Development Center (SHDC).

SAS software was used for statistical analysis. The results of this trial will be published to the public.

## Trial status

The trial was registered at ClinicalTrials.gov on 13 November 2020 (identifier ChiCTR2000036515). We started recruitment in February 2021, and it was expected to be completed in November 2022. All items from the World Health Organization Trial Registration Data Set are shown in Table 2.

**Table 2:** WHO trial registration data

Data category	Information
Primary registry and trial identifying number	ClinicalTrials.gov ChiCTR2000036515
Date of registration in primary registry	13 November 2020
Secondary identifying numbers	ChiMCTR2000003660
Source(s) of monetary or material support	Clinical Research Plan of SHDC (No. SHDC2020CR1051B)
Primary sponsor	Hongsheng Zhan
Secondary sponsor(s)	N/A
Contact for public queries	Hongsheng Zhan, Ph.D., Professor, E-mail address: shgsyjs@139.com
Contact for scientific queries	Hongsheng Zhan, Ph.D., Professor Shi's Center of Orthopedics and Traumatology (Institute of Traumatology, Shuguang Hospital), Shuguang Hospital Affiliated to Shanghai University of Traditional Chinese Medicine, Shanghai, China
Public title	A multicenter clinical research on the treatment of lumbar intervertebral disc herniation with nerve root adhesion with integrated traditional Chinese and minimally invasive spine surgery
Scientific title	Study on the efficacy and safety of the combination of Shi’s manual therapy and percutaneous endoscopic lumbar diskectomy for lumbar disc herniation with radiculopathy: Study Protocol for a Multicenter Randomized Controlled Trial
Countries of recruitment	China
Health condition(s) or problem(s) studied	lumbar disc herniation with radiculopathy
Intervention(s)	Shi’s manual therapy (SMT)
	percutaneous endoscopic lumbar discectomy (PELD)
Key inclusion and exclusion criteria	Ages eligible for study: 20 to 70 years old; Sexes eligible for study: both; Accepts healthy volunteers: no
	Inclusion criteria: meet the diagnostic criteria of lumbar disc herniation with radiculopathy; ODI ≥ 10; The course of the condition is more than 3 months and has not improved with conservative therapy for more than 6 weeks; Voluntarily participated in this trial and signed the informed consent form.
	Exclusion criteria: Tuberculosis, cancer, severe osteoporosis, acute infectious diseases, acute suppurative inflammation, severe cardiovascular, cerebrovascular, liver, kidney, hematopoietic, digestive system diseases or mental disorders.; Definite lumbar segmental instability or developmental lumbar spinal stenosis; Current pregnancy or is prepared to get pregnant; Received other treatment at the same time; Those who participated in other clinical trials within 3 months.
	Rejection criteria: The researcher considered unsuitable for participating in the trial, (for example, unable to correctly understand the questionnaires, inconvenient to follow-up for living in other provinces); The subjects disobeyed the trial plan and combined with other drugs; Serious adverse reactions; The patients’ complaints continued to increase, which proved that trial participation was not suitable.
Study type	Interventional
	Allocation: randomized; Intervention model: parallel assignment; Masking: unblinded treatment and blinded outcome assessment
	Primary purpose: efficacy and safety
Date of first enrolment	24 February 2021
Target sample size	510
Recruitment status	Recruiting
Primary outcome(s)	Recurrence rate of herniation at the same level and on the same side (time frame: 1-year; not designated as safety issue)
Key secondary outcomes	ODI Index; VAS of lumbar and lower limb pain; Straight leg raising angle of the affected side (time frame: 1-year; not designated as safety issue)

## Data Availability

Not applicable.

## Abbreviations

SMT: Shi’ manual therapy
PELD: Percutaneous endoscopic lumbar discectomy
LDH: Lumbar disc herniation
RCT: A randomized controlled trial
TCM: Traditional Chinese medicine
NSAID: The nonsteroidal anti-inflammatory drug
ODI: Oswestry Disability Index
SF-36v2: Short Form 36 version 2
VAS: The visual analog scale
MRI: Magnetic resonance imaging
CT: Computed tomography
EMG: Electromyography
AP: Anteroposterior radiographic views
